# Impact of the Mk VI SkinSuit on skin microbiota of terrestrial volunteers and an International Space Station-bound astronaut

**DOI:** 10.1038/s41526-017-0029-5

**Published:** 2017-09-07

**Authors:** Richard A. Stabler, Helena Rosado, Ronan Doyle, David Negus, Philip A. Carvil, Juan G. Kristjánsson, David A. Green, Rafael Franco-Cendejas, Cadi Davies, Andreas Mogensen, Jonathan Scott, Peter W. Taylor

**Affiliations:** 1 Department of Pathogen Molecular Biology, London School of Hygiene and Tropical Medicine, London, UK; 2 School of Pharmacy, University College London, London, UK; 3 Division of Infection and Immunity, University College London, London, UK; 4 Centre of Human & Aerospace Physiological Sciences, King’s College London, London, UK; 5 European Astronaut Centre, European Space Agency, Köln, Germany

## Abstract

Microgravity induces physiological deconditioning due to the absence of gravity loading, resulting in bone mineral density loss, atrophy of lower limb skeletal and postural muscles, and lengthening of the spine. SkinSuit is a lightweight compression suit designed to provide head-to-foot (axial) loading to counteract spinal elongation during spaceflight. As synthetic garments may impact negatively on the skin microbiome, we used 16S ribosomal RNA (rRNA) gene amplicon procedures to define bacterial skin communities at sebaceous and moist body sites of five healthy male volunteers undergoing SkinSuit evaluation. Each volunteer displayed a diverse, distinct bacterial population at each skin site. Short (8 h) periods of dry hyper-buoyancy flotation wearing either gym kit or SkinSuit elicited changes in the composition of the skin microbiota at the genus level but had little or no impact on community structure at the phylum level or the richness and diversity of the bacterial population. We also determined the composition of the skin microbiota of an astronaut during pre-flight training, during an 8-day visit to the International Space Station involving two 6–7 h periods of SkinSuit wear, and for 1 month after return. Changes in composition of bacterial skin communities at five body sites were strongly linked to changes in geographical location. A distinct ISS bacterial microbiota signature was found which reversed to a pre-flight profile on return. No changes in microbiome complexity or diversity were noted, with little evidence for colonisation by potentially pathogenic bacteria; we conclude that short periods of SkinSuit wear induce changes to the composition of the skin microbiota but these are unlikely to compromise the healthy skin microbiome.

## Microbiology: gravity-mimicking suit safe for skin bacteria

A compression garment that applies gravity-like pressure to the skin alters the composition of skin microbes, but not in a dangerous manner. A team led by Peter Taylor from University College London, UK, characterised the bacterial skin communities at dry and moist body sites of five Earth-bound volunteers before and after wearing the Mk VI SkinSuit, which creates a pressure loading system that simulates gravity’s effects. 8 h in the SkinSuit changed the skin microbiota at the genus level but had little to no impact in community structure. The researchers observed more dramatic changes in one astronaut who wore the garment on the International Space Station. However, the microbial makeup reverted back to pre-flight profiles upon the astronaut’s return to Earth. The findings suggest that short-term SkinSuit wear is unlikely to compromise bacterial skin health.

## Introduction

The microgravity environment encountered in Low Earth Orbit aboard the International Space Station (ISS) induces physiological deconditioning due to the absence of gravity loading, such as a loss of bone mineral density of 1–2% per month^1^ and atrophy of skeletal muscle, in particular of the lower limb.^2, 3^ In addition, astronauts experience a lengthening of the spine of up to 7 cm, back pain and some show evidence of intervertebral disc damage post flight.^4^ While exercise reduces musculoskeletal conditioning, it appears not to alleviate spinal elongation and back pain. Provision of compressive forces along the axial (Gz) body axis was first achieved by the Russian Pingvin suit which imposed loading through bungee cords wrapped around the shoulders and feet while tethered to a central belt.^5^ However, the two-point 40 kg loading does not reproduce the graduated effect of gravity, where there is little loading at the shoulders and the full 1 G effect is experienced only at the soles of the feet. As a result, cosmonauts find the suit too uncomfortable and thus it is rarely used, while its effectiveness upon spinal elongation has yet to be defined. Furthermore, the Pingvin suits are not washable and become odoriferous,^6^ suggesting increases in unwanted skin bacteria at the suit-skin interface.^7^

The Mk VI SkinSuit^8^ is a lightweight (<500 g) compression garment designed to replicate the gradual increase in Gz loading down the body in a manner more consistent with that experienced on Earth, thereby promoting comfort and efficacy. The SkinSuit comprises a bidirectional weave that imposes weight-bearing-like loading by gradually increasing tension in the Gz axis fibres, with a low circumferential tension to prevent suit slippage. While the principle is based on the gravity loading countermeasures Skinsuit^8^ the design, loading regime and materials used in the Mk VI SkinSuit differ substantially, with superior comfort and a reproducible, albeit lower axial loading of approximately 0.16 Gz. Nevertheless, such loading has been shown to be sufficient to attenuate hyper-buoyancy dry floatation (HBF)-induced spinal elongation while being compatible with ambulation and cycling up to 75% VO_2max_^9, 10^ as required for ISS implementation. The main body of the SkinSuit, and the region in contact with the skin, is comprised of Elast 200-Second, a composite of polyamide/nylon and elastane; studies with related synthetic fabrics indicate that such materials may be prone to sweat-induced problems of odour generation, discolouration and loss of textile performance.^11^

The human body is colonised by a large and diverse community of microorganisms, the microbiota, that engage in complex and poorly understood interplay with the host. Predominantly bacteria residing on the skin and in the oral cavity, nasal passages and urogenital and gastrointestinal tracts, they influence the development and function of key physiological processes, including the orchestration of the mucosal immune response.^12, 13^ The skin is an extensive ecosystem with a range of microhabitats that supports a large and diverse range of bacteria and other microorganisms. The advent of massively high throughput sequencing, culture-independent methods for investigation of bacterial populations on human skin has revealed the complexity and diversity of the skin microbiota, with more than 200 bacterial genera and 1000 species occupying various sebaceous, moist and dry sites.^14^ Space farers on extended missions^15, 16^ and individuals who have remained non-ambulatory for a significant amount of time^17, 18^ suffer changes to their natural bacterial skin microbiota, with increases in potentially harmful bacteria and decreases in protective commensals. Further, significant reductions in the numbers of beneficial bacterial components of the microbiota have been observed in astronauts preparing for flight and attributed to pre-launch stress.^19^

To develop and validate 16S rRNA gene amplicon procedures for sampling of skin bacteria and to determine the impact of SkinSuit wear on the skin microbiota we evaluated fluctuations in the bacterial microbiome from four discrete body sites of volunteers undergoing HBF studies, using DNA amplification and sequencing of the hypervariable V3-V4 rRNA region of the bacterial genome. We then evaluated changes to the skin microbiota of an ESA astronaut during a 10-month pre-flight training period, during an 8-day mission aboard the ISS (when SkinSuit was worn for 6–7 h periods on two consecutive days) and a 1-month recovery period. Qualitative alterations in the microbiota, without loss of the complexity and diversity of the bacterial skin population, were associated with changes in astronaut location during pre-flight training. The terrestrial and flown studies indicated that short term SkinSuit wear is unlikely to deleteriously impact on the bacterial population of the skin.

## Results

### Validation of skin sampling procedure

The full extent of microbial diversity across all skin layers is best determined by skin scraping or punch biopsy^20^ but these invasive methods were considered impractical for the purposes of the current study. Five locations on the skin of a single healthy volunteer were sampled using various moist and dry commercially available cotton and rayon swabs and by application of autoclave and microporous tape to the skin surface. We confirmed that more extensive profiling of bacterial skin populations are obtained using culture-independent (16S rRNA gene amplicon) procedures rather than bacterial culture that uses a variety of selective and non-selective solid media; many bacteria do not grow, or grow only slowly, on laboratory media and many are not identifiable by culture techniques.^21, 22^ 16S rRNA gene amplicon profiling was therefore employed throughout this study. Neither tape method yielded consistent 16S rRNA gene amplicon data. The most reproducible data was obtained with rayon-tipped Sterilin F155CA swabs moistened with phosphate-buffered saline; these were used for the HBF study. For the astronaut study we employed ISS certified Dacron-tipped swab rinse kits containing 5 ml saline; these performed in identical fashion to the Sterilin swabs.

### Hyper-buoyancy floatation (HBF) human volunteer study

**Fig. 1:**
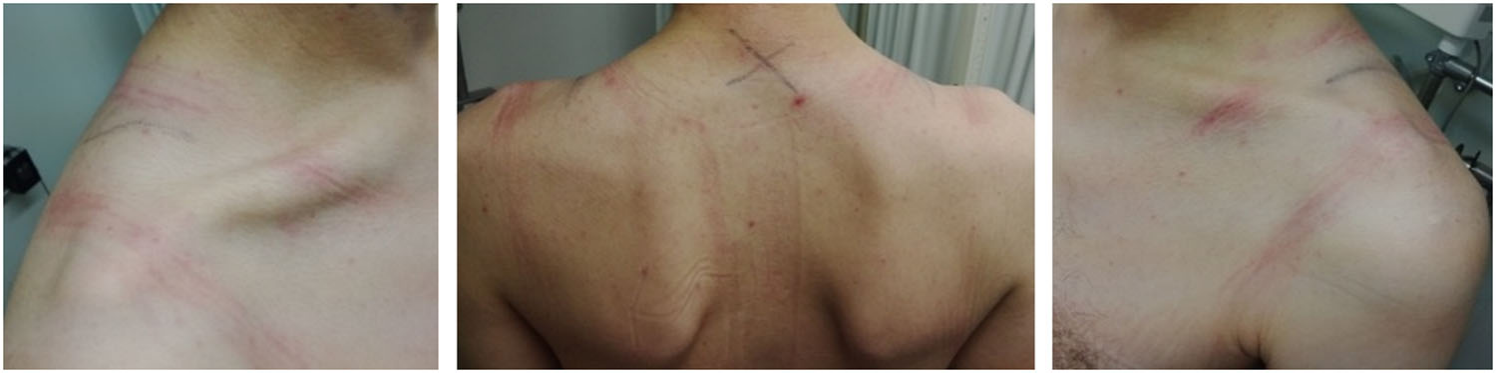
Some skin marking and skin irritation (above) was evident after SkinSuit wear by some subjects following vigorous exercise. No exercise was undertaken during the HBF volunteer study, although we anticipate that prolonged exercise will be undertaken by astronauts during flight as development of the Mk VI SkinSuit progresses

Five healthy male volunteers (♂ = 5; 28.4 ± 5.9 yr; 182.6 ± 9.7 cm; 77.3 ± 8.3 kg) were provided with a custom-fabricated Mk VI SkinSuit, which necessitated detailed sizing and fibre load determination for each subject. Although each customised SkinSuit was considered to be comfortable and did not restrict movement, extended wear can induce some minor skin irritation after exercise, as is evident following maximal cycle ergometry in 1 Gz (Fig. 1). However, in the current volunteer study no exercise was performed: following familiarisation, including practice donning and doffing the SkinSuit, each individual lay on the HBF for 8 h in gym clothing in one session, and then again for 8 h in their SkinSuit on a subsequent day, no more than 1 month later. Following familiarization, including practice donning and doffing the SkinSuit, each individual lay on the HBF platform for 8 h in gym clothing in one session and again for 8 h in their SkinSuit on a subsequent day no more than 1 month later. HBF is a novel ground-based analogue of unloading where the immersed subject is suspended upon a hypersaline water mass While remaining dry; this system is similar to dry immersion^23^ but facilitates improved access to the volunteer and may prove more suitable for musculoskeletal unloading studies. Bacterial microbiomes from two sebaceous (chest; lower back) and two moist (armpit; groin) skin sites were determined before and after both periods of HBF.

**Fig. 2:**
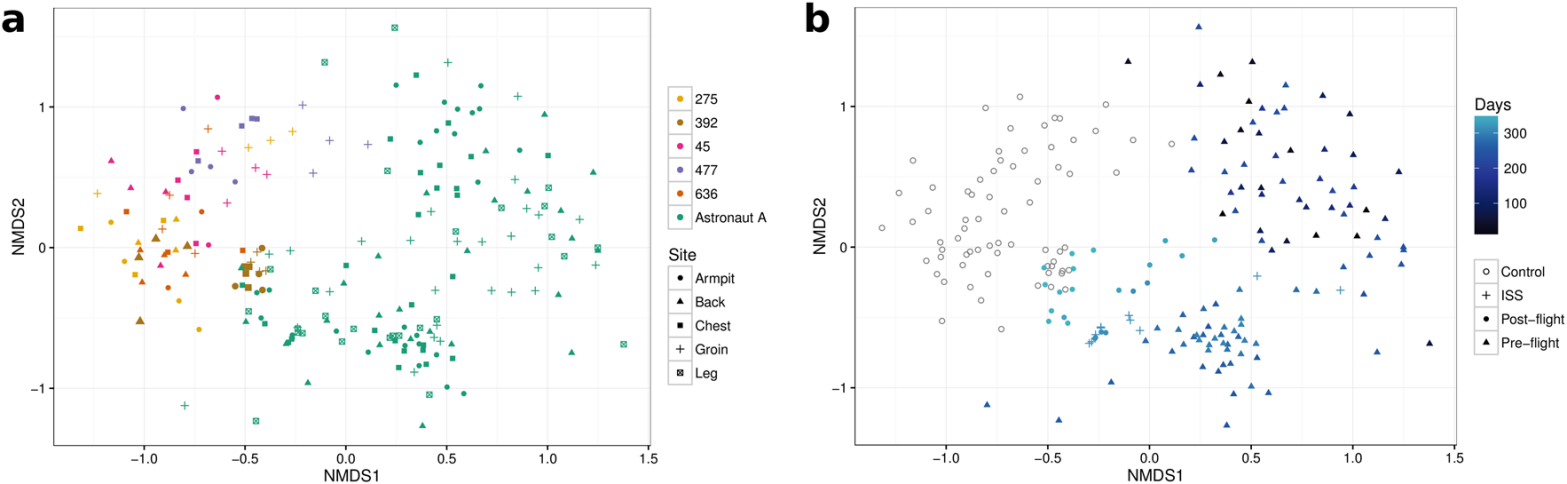
Cluster analysis of dissimilar bacterial diversity in skin samples from dry immersion volunteers and Astronaut A as visualised by non-metric multidimensional scaling (NMDS). Data from Astronaut A was analysed in comparison to data from volunteers with respect to sampling site (**a**) and phase of mission (**b**)

We sequenced 31,015,421 Illumina reads from the volunteer samples, generating 9,952,712 individual sequences from the V3/V4 region of bacterial 16S rRNA genes. Data were discarded from V3/V4 regions that failed to match a closed Greengenes 16S rRNA gene database (http://greengenes.lbl.gov); 36.7% (3,651,743) passed the NINJA-OPS quality filter. The NINJA-OPS bioinformatics pipeline^24^ was also used to assign sequences to 1312 operational taxonomic units (OTUs). Seven of 76 of immersion study swabs yielded no bacterial DNA and were therefore excluded from the study. With some DNA samples (3/204; 1.5%), less than 3200 reads were obtained and these were not analysed further. Data were filtered to remove extremely rare bacterial species (e.g., OTUs found once in only one sample) and experimental errors (e.g., chimeras generated during PCR amplification). The taxonomic distribution of OTUs of each sample is shown in Interactive Data file S1 (Supplementary Information). Alpha and beta diversity^25^ (diversity within samples and diversity between samples, respectively) of OTUs from all sequences that passed the filters were analysed by determination of sample clustering using principal component analysis (PCoA) based on unweighted Unifrac distances (Fig. 2). A minimum count fraction mean of 0.0001 enabled identification of 18/20 bacteria in the mock community at genus level and was used for analysis of all samples. In line with current understanding of the skin microbiome,^26–28^ variation between individuals and between different skin sites on each individual volunteer was evident. Rarefaction curves^29^ were constructed to determine the level of species richness associated with all sampled body sites; the data indicated that HBF with either gym clothing or SkinSuit had negligible impact on the diversity of bacterial species associated with the skin microbiome based on a minimum of ~3000 reads (Supplementary Fig. S1). The large error bars were due to 16S rRNA gene amplicon heterogeneity (Fig. 2) and consequently differences between different body sites and between individual volunteers did not attain levels of statistical significance. Due to low Good’s coverage scores, visualisations of alpha diversity rarefaction curves were produced to manually check sequencing depth. Curves representing those samples with the lowest Good’s coverage scores showed plateauing of the curve and suggested that current sequencing depths were adequate to capture community diversity (Supplementary Fig. S2). Maximum species diversity was achieved with ~2000 reads, indicating that more intensive sampling is likely to yield few additional species.

**Fig. 3:**
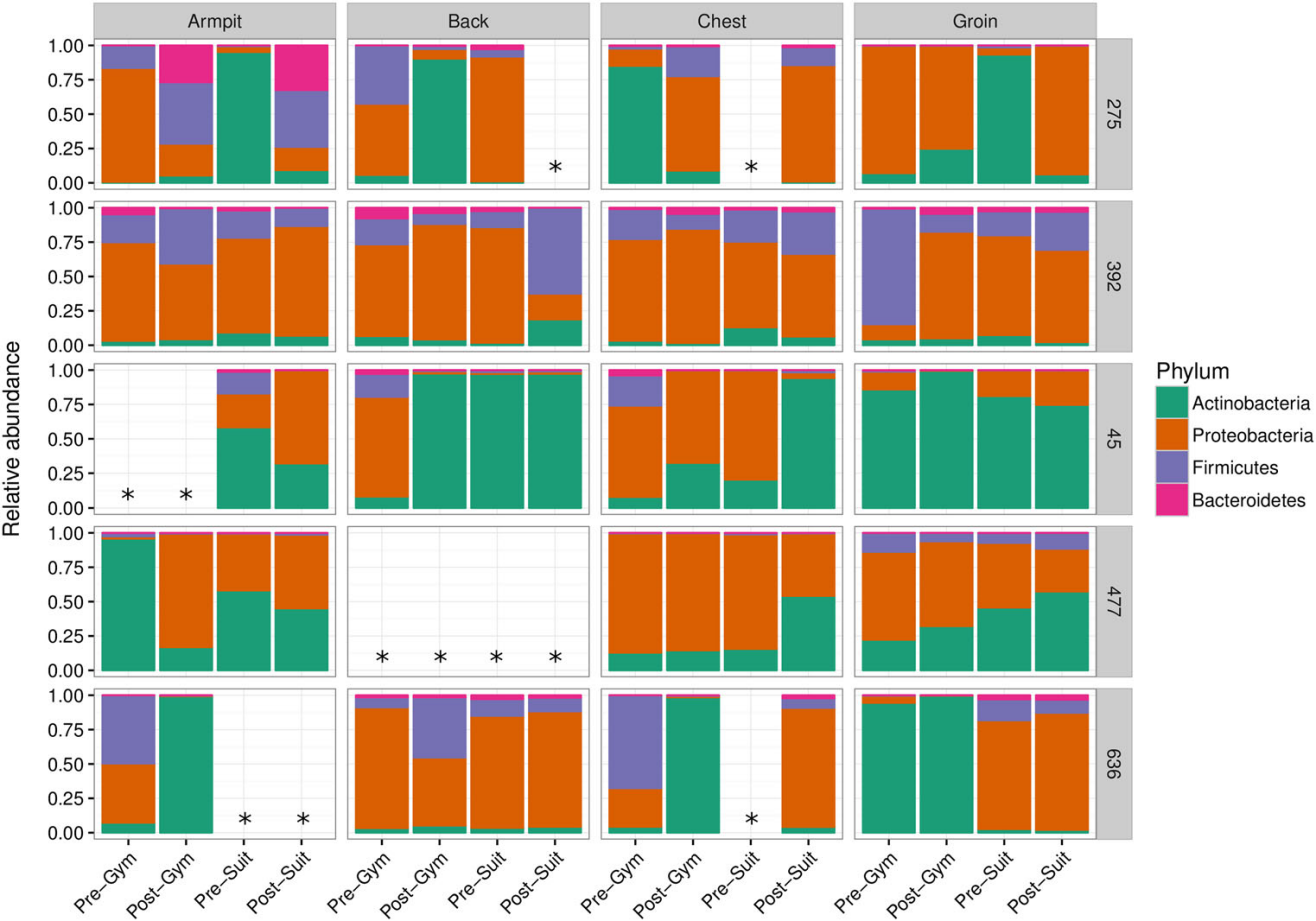
Bacterial phyla recovered from the armpit, back, chest and groin of dry immersion volunteers. Some data was discarded due to failure of samples to pass quality filters, as detailed in the Results section

**Fig. 4:**
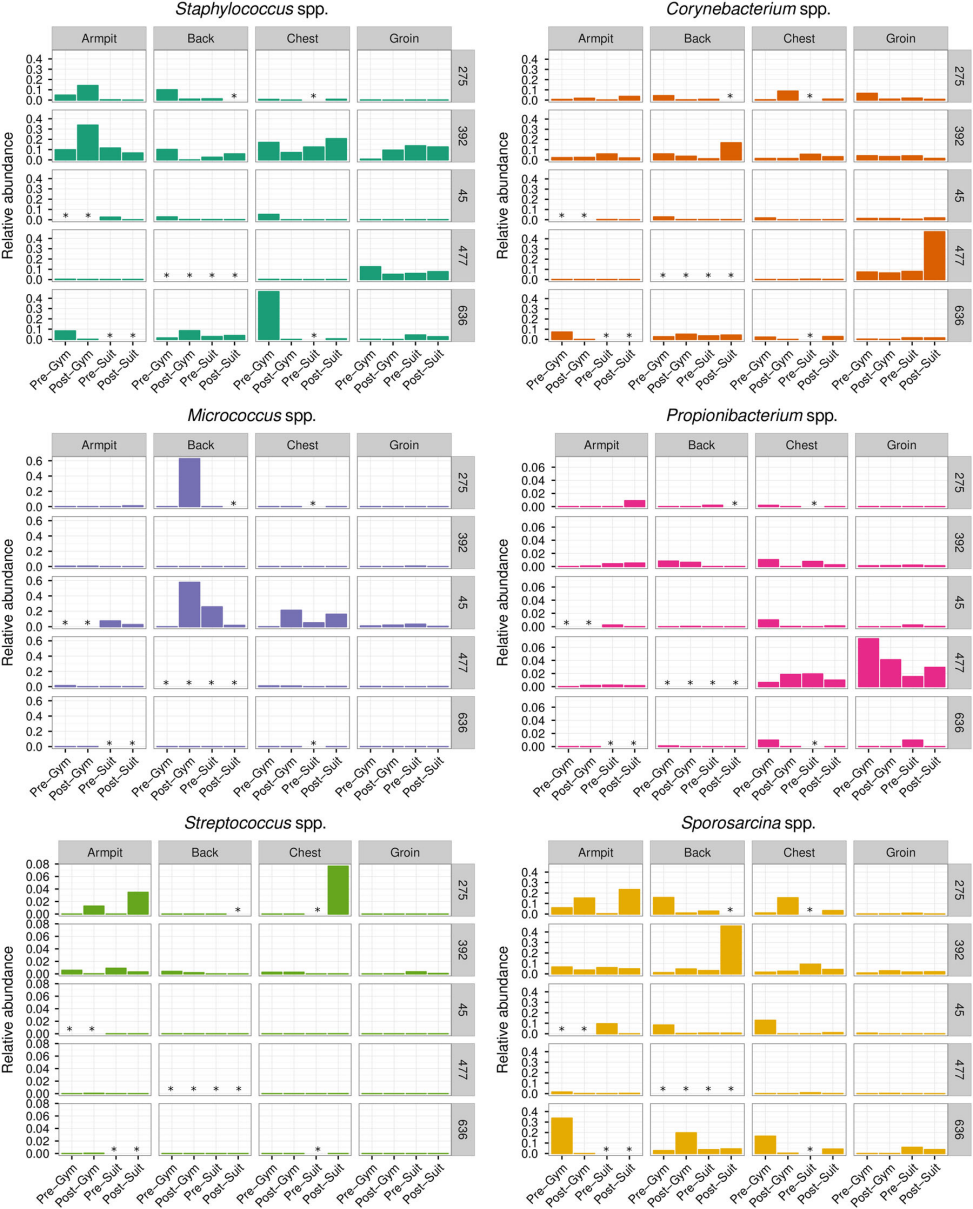
Relative abundance of key bacterial genera recovered from dry immersion volunteer samples. *Staphylococcus* spp, *Corynebacterium* spp, *Micrococcus* spp, *Propionobacterium* spp and *Streptococcus* spp are ubiquitous components of the skin microbiota of healthy human adults. *Sporosarcina* spp are shown as examples of an environmental genus strongly associated with the skin of volunteers over the course of this study

The healthy skin microbiota is rich and diverse but the majority of bacteria belong to four phyla: Actinobacteria, Firmicutes, Proteobacteria and Bacteroidetes.^14, 30^ Figure 3 shows the bacterial populations at the phylum taxonomic level recovered by swabbing of the armpit, back, chest and groin of each volunteer and confirms the high degree of variability between individuals. It has been established that bacterial diversity is lower in sebaceous sites such as chest and back in comparison to moist sites such as armpit and groin, suggesting that not all bacteria are able to tolerate sebaceous secretions.^14^ Sebaceous sites were dominated by Actinobacteria (e.g., volunteer #45; back) or Proteobacteria (#392; chest and back) but dominant populations of Acidobacteria were found in chest and back samples from #636 and were ubiquitous in all samples from #392. Both moist sites from #392 yielded sequences from the four established phyla and also Acidobacteria but in volunteers #45 and #477 Proteobacteria and Actinobacteria were dominant. Thus, at the phylum level there was a high degree of variability amongst comparable young, healthy males and this was reflected at the class, order, family and genus levels of the taxonomic hierarchy (Interactive Data file S1). At the genus level, there was considerable variation between individuals and between body sampling sites from the same individual (Fig. 4; Interactive Data file S1). Genera commonly associated with the skin microbiome, such as *Staphylococcus*, *Propionibacterium*, *Corynebacterium*, *Streptococcus* and *Micrococcus*, were frequently encountered and we also regularly identified environmental bacteria that included *Pseudomonas*, S*porosarcina*, *Caulobacter*, *Novosphingobium*, *Agrobacterium* and *Actinomyces*. HBF in both gym kit and SkinSuit altered the bacterial profile of the skin but there was generally no diminution in the complexity and diversity of the microbiome (Interactive Data file S1). In one individual, #477, the lipophilic Actinobacteria *Propionibacterium* and *Corynebacterium*, often present in sebaceous regions of the skin,^14^ were more prevalent in the groin than with other volunteers and persisted throughout the investigation period (Fig. 4).

### Skin sampling of Astronaut A

**Fig. 5:**
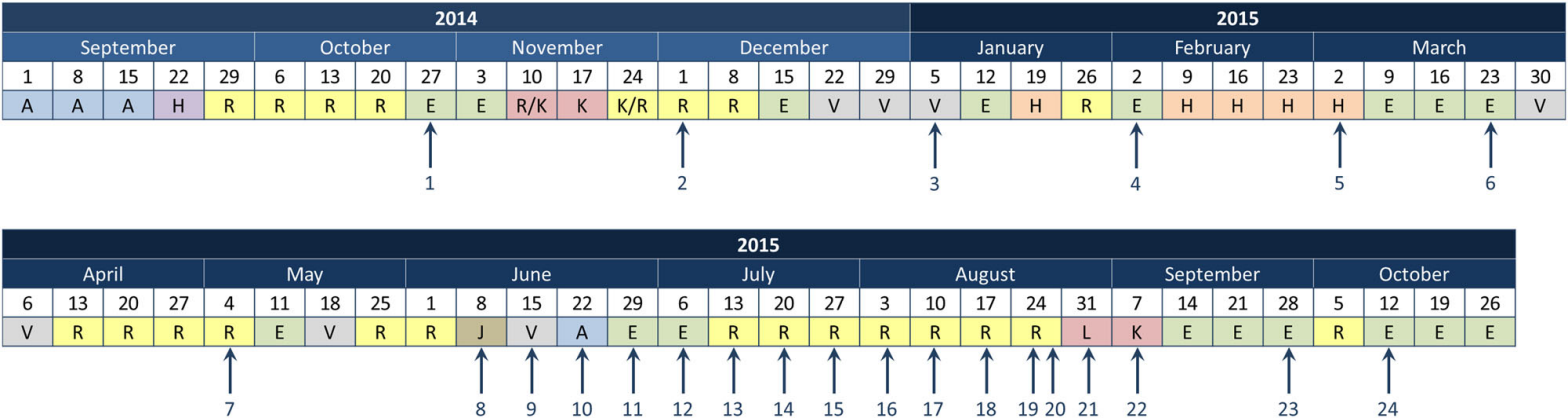
Skin sampling of Astronaut A was undertaken at regular intervals (indicated by arrows) prior to, during and after an 8-day mission to the ISS in September 2015. SkinSuit was worn for two 8-h periods aboard the ISS. Locations during the study period are indicated: R, Russia; E, Europe; V, vacation; K, Kazakhstan; H, Houston; J, Japan; A, USA excluding Houston; L, Launch period. Boxes below month represent weeks

Determination of the bacterial skin microbiota at five body sites (armpit, back, chest, groin and a dry site on the leg) began in October 2014, and these sites were sampled with increasing regularity to launch on 2nd September 2015; swabbing was undertaken aboard the ISS and after return to Earth on 12th September 2015 up to mid-October 2015 (Fig. 5). All samples were taken before morning shower and use of large quantities of body lotion or antiperspirant was discouraged. Samples were collected and analysed together as a single batch: 24,308,718 individual sequences from the V3/V4 region were generated from 95,850,353 Illumina reads and 44.6% (10,880,148) passed the NINJA-OPS quality filter. The NINJA-OPS bioinformatics pipeline was used to assign sequences to 2433 OTUs. The taxonomic distribution of OTUs of each sample is shown in Interactive Data file S2 (Supplementary Information).

**Fig. 6:**
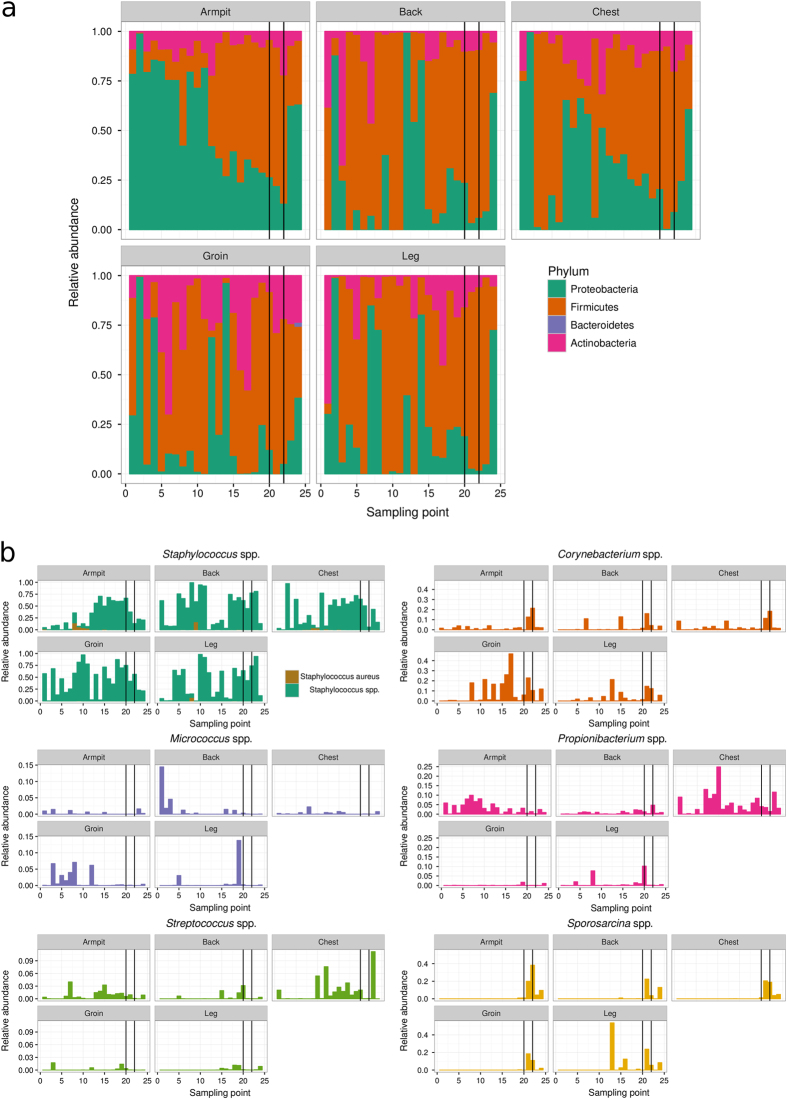
16S ribosomal RNA gene amplicon analysis of bacterial skin microbiota of Astronaut A during period 2014–5 **a** Bacterial phyla recovered from the armpit, back, chest, groin and leg. **b** Relative abundance of key bacterial genera recovered during the study period

PCoA performed on OTU data from all sequences that passed the filters indicated a high degree of beta-diversity between body sites during the different phases of the mission; there was a clear difference in clustering between samples from Astronaut A and those of the five HBF volunteers (Fig. 2). This was confirmed as statistically significant by application of both ANOSIM (*p* < 0.001) and PERMANOVA (*p* < 0.001). Canonical Correspondence Analysis (CCA) showed this was due to higher abundance of *Staphylococcus*, *Propionibacterium* and *Corynebacterium* in the samples from Astronaut A compared to the volunteers and to higher abundance of *Micrococcus* and *Paracoccus* in the volunteers (Supplementary Fig. S3). Rarefaction curves indicated some differences in species richness (alpha-diversity) from the five body sites but these did not reach levels of statistical significance (Supplementary Fig. S4). As with the volunteer samples, large error bars were most likely due to 16S rRNA gene amplicon heterogeneity. Near maximal (>95%) species diversity was achieved with >3000 reads. In all, a total of 11 phyla were detected, with Firmicutes (43.1%), Proteobacteria (40.1%), and Actinobacteria (14.6%) the most frequently observed (Fig. 6). Bacteroidetes were also present in armpit (2.4%), chest (0.8%), leg (0.7%) and back (0.2%) samples, and Acidobacteria was documented at the armpit site (2.7%). Wet sites tended to have more Proteobacteria (36.6%); Firmicutes predominated at sebaceous (47.9%) and dry (47.9%) sites.

The complete data set of bacteria identified at phylum, class, order, family and genus levels in all 55 Astronaut A samples can be viewed in Interactive Data file S2. Initially the armpit was dominated by Proteobacteria (>75%); however, during pre-flight training these become less frequent (25%) as launch approached due to an expansion of the Firmicute population (Fig. 6). During the ISS mission, the Proteobacteria were further depleted due to Firimcute expansion but they rapidly regained niche dominance on return to Earth. A similar population profile was identified with chest and, to a lesser extent, back samples. Groin and leg showed similar microbiota profiles, generally dominated by Proteobacteria or Firmicutes; three groin samples temporarily showed Actinobacteria to be the dominant phylum. Aboard the ISS, Proteobacteria were replaced in the main by Firmicutes in groin and leg samples to mirror more closely the armpit, back and chest but Proteobacteria re-established themselves in the groin and leg as the dominant microbiome components on return to Earth. In these samples Firmicute expansion was due in part to an increase in the staphylococcal, and to a lesser extent streptococcal, population. These changes were confirmed using CCA with Proteobacteria genera such as *Escherichia* and *Shigella* separating pre-flight samples and Firmicute genera such as *Sporosarcina* responsible for the greatest discriminatory effect for ISS and post-flight samples. Overall, staphylococci comprised the most abundant bacterial genus and were dominant in all five body sites over long periods of the study. The dominant genera *Staphylococcus* and S*treptococcus* contain both harmless commensular and pathogenic species; comparison of staphylococcal OTUs with those in the Greengenes 16S database^31^ enabled their assignment to the potentially pathogenic species *Staphylococcus aureus* and to *Staphylococcus hominis*, a harmless common resident of the skin (Supplementary Table S1). *S. hominis* was the most commonly encountered bacterium in the microbiota from Astronaut A and we detected *S. aureus* with low frequency during the pre-flight phase (Fig. 6; Supplementary Table S1). We also identified *Staphylococcus warneri* with high frequency; this coagulase-negative staphylococcal bacterium is a common saprophyte on found on skin from ~50% of the healthy adult population but it has emerged as an uncommon cause of serious infection in the last two decades in individuals with a predisposing condition.^32^

Significant numbers of Actinobacteria and Firmicutes were also detected with no obviously dominant actinobacterial genera linked to the expansion of this phylum, although *Corynebacterium* spp were present in samples taken during the late training phase with up to 40% expansion at all sampled body sites during the ISS visit. These returned to levels found during the pre-flight period on return to Earth. This data provide strong evidence for changes in the skin microbiome (Interactive Data file S2; Fig. 6) in response to changes in geographical location (Fig. 5). Unusually, *Sporosarcina* spp, environmental bacteria most commonly associated with soil contaminated with urine such as cow pastures^33^ and finding utility as biomineralising agents for construction materials,^32, 34^ were identified in occasional pre-flight leg swabs but appeared as a dominant genus in samples from all five body sites taken on board the ISS. Recovery decreased markedly on return to Earth (Interactive Data file S2; Fig. 6).

## Discussion

It is well established that there is a wide diversity of bacteria within and between distinct areas of the skin surface and that the composition of the human skin microbiome is shaped by biogeography, individuality and wellbeing.^26–28, 35^ Thus, the diverse skin microenvironments that exist both within and between individuals contribute to the profile and community organisation of the skin microbiota which in itself is subject to modification over time through interactions with the external environment. These influences include usage patterns,^36^ washing procedures and attire,^7, 11^ particularly when clothing is in constant contact with the skin.^11^ As residents of the outermost layer of the body surface, skin bacteria may in turn contribute to the microbial composition of the immediate environment, particularly in closed habitats such as the ISS. Surfaces on board the Russian segment of the ISS were initially colonised with skin-associated genera such as *Staphylococcus*, *Micrococcus*, *Bacillus* and *Streptococcus*, indicating that the primary seeding of ISS contamination was due to shedding of skin by crew.^37^ With continuous human occupancy, a diverse contaminating microbial population has developed over a relatively short period of time and includes over seventy distinct bacterial species as well as biodestructive and potentially pathogenic fungi; ISS bacterial contamination continues to be dominated by core components of the skin microbiota.^38–40^

All five healthy volunteers exhibited distinct individual microbial signatures at each body site. Changes in the composition of the bacterial skin community were associated with wearing of both gym kit and the Mk VI SkinSuit for 8-h periods and is in line with previous data recorded using 16S rRNA gene methods that wearing of sports textiles directly influences the nature of the skin microbiota.^11^ As the period of wear was relatively short (8 h), changes to the microbiota may be due to some degree of modest bacterial proliferation of pre-existing populations. This issue can only be resolved by determination of the impact of longer periods of garment wear on the skin microbiota. The bacterial communities that could be recovered from the skin of volunteers were highly complex with a heterogeneous mix of genera representing the four predominant phyla as well as other phyla less frequently associated with the skin (Interactive Data file S1). Although the composition of these communities was affected by dry immersion there was no diminution in their complexity or diversity and no obvious enrichment for genera that might contain pathogenic representatives. The factors which maintain the taxonomic and functional diversity of the skin microbiota are not well understood but a shift towards a less diverse microbiota is associated with disease.^41^ Space flight may also cause deleterious changes in the microbiota: in studies undertaken before the advent of massively high throughput 16S rRNA gene amplicon sequencing tools for evaluation of complex microbial communities, Soviet scientists reported that adverse shifts in the skin and respiratory microbiota of crew occurred aboard short (up to 5 days) Vostok missions and longer (up to 24 days) Soyuz flights^16^ although major changes in bacterial composition were not evident in early American (Apollo) flights.^42^

There is evidence that the composition of the bacterial microbiota is modulated during preparation for flight and the post-flight recovery period, as well as during the flight itself.^19, 43^ For this reason, we collected skin swabs from Astronaut A beginning 1 year before projected launch, during the period aboard the ISS and for 1 month after return to Earth. The skin microbiota, as expected, varied with respect to body site and we noted abrupt changes in the community profile triggered by change of location as detailed in Fig. 5. The difference in phylogenetic distances between bacterial communities was particularly evident during the period aboard the ISS but reverted to a profile similar to pre-flight composition on return to Europe from Kazakhstan/Russia (Fig. 2). As with the HBF volunteers, changes in bacterial profile were not accompanied by any reduction in community complexity and they continued to reflect a healthy microbiota with near-absence of potentially pathogenic bacteria. As the Staphylococcaceae are the dominant family aboard the ISS,^44^ with around 85% of surface samples yielding these bacteria,^37^ we assigned the OTUs to species within this group (Fig. 5; Supplementary Table S1). Potentially troublesome *S. aureus* was found as a minor component of the microbiota in a limited number of samples during pre-flight training and was considered normal: around 20–30% of healthy human subjects are colonised persistently with *S. aureus*, 30% intermittently and 50% rarely or never colonised.^45, 46^ Thus, colonisation *by S. aureus* is not synonymous with infection and may be better considered as a normal component of the nasal and skin microbiota.

Microbiomes from Astronaut A and all HBF volunteers yielded a variety of bacteria that are generally considered to be environmental microorganisms (Interactive Data files S1 & S2) and we consider it likely that these are transient members of the bacterial skin community present due to exposure to urban or rural sources of contamination. Prominent amongst these were bacteria of the genus *Sporosarcina*; they were recovered as significant microbiome components from armpit, back and chest of four of the five volunteers (Fig. 4). They were also found on occasion in leg swabs taken pre-flight from Astronaut A but dominated samples from all five body sites aboard the ISS. On return, *Sprorosarcina* populations rapidly subsided. Although direct evidence is lacking, it seems reasonable to assume that these bacteria are SkinSuit contaminants, as their appearance coincides with SkinSuit wear. The individually customised suits are manufactured under standard factory conditions and not in the ultraclean facilities used to fabricate spacecraft, so it is possible that *Sporosarcina* contamination occurred during manufacture, although we did not initiate attempts to recover *Sporosarcina* from samples taken from SkinSuit. Interestingly, *Sporosarcina* has been examined at the NASA Ames Research Center in Moffett Field, California as a potential catalyst for the production of bricks and mortar for future human settlers on Mars;^47^ Astronaut A did not visit this facility during the pre-flight period. Recently, *Sporosarcina* spp have been found to be major components of a unique community of microbes harboured in a particulate-controlled, restricted access cleanroom at NASA Jet Propulsion Laboratory;^48^ they were able to survive rigorous cleaning procedures designed to eliminate more recognised potential contaminants.

Our initial examination of the impact of SkinSuit wear on skin microbiota had a number of important limitations which we will aim to eliminate in future studies. The size of the volunteer cohort was limited by the number of subjects willing to participate in the relatively short (8 h) but rigorous hyper-buoyancy study, by limited availability of customised SkinSuits (due to their high cost and limitations on manufacture) and by the fact that we included only males in the metagenome study. Seven of 76 swabs from the immersion study yielded no detectable bacterial DNA. These samples were excluded from the study and did not influence the conclusions drawn. We have no explanation for these anomalies; sampling by the volunteers was directly supervised by an investigator and no inconsistencies in sampling technique were flagged during the course of the study. The nature of both the volunteer and astronaut studies precluded the acquisition of a full set of technical and biological replicates; we will attempt to overcome these difficulties in future SkinSuit development activities.

No obvious odours were reported by any participant following SkinSuit wear. It has been reported^7^ that unpleasant odours may arise following intensive exercise and a trained odour panel identified significant differences between synthetic and cotton garments, with the odour from polyester less pleasant and more intense.

## Materials and methods

### Terrestrial volunteers

Five young healthy male participants gave written, informed consent to participate in the study, which was approved by the ethical committee of King’s College London. Participants had no history of neurological, cardiorespiratory, musculoskeletal conditions (including chronic back pain) and/or psychological disorder. Participants were asked to abstain from alcohol, vigorous exercise and any pharmaceuticals for 24 h prior to testing and completed a back pain questionnaire (ISS pre-flight questionnaire, ESA). Subjects completed two counterbalanced experimental conditions by laying supine for 8 h when buoyant on a waterbed (Hyper-Buoyant Floatation; HBF) part-filled with (300 l) hypersaline solution (with a high specific density) contained within a 2 × 1.2 m medium density fibreboard frame. In one condition subjects wore normal gym clothing and, in the other, a newly fabricated, previously unworn Mk VI SkinSuit. The thermal neutrality of participants was attained by heating the HBF water to 34 °C using an element placed beneath the bed. Volunteers collected their own skin samples. A communal briefing together with a quick reference guide (including the swabbing method) was provided to ensure reproducibility of the sampling method and to minimise bias as much as possible. Sampling was performed with participant consent under direct supervision by an investigator. Methods were performed in accordance with relevant regulations and guidelines; volunteer gave consent for published image.

### Skin sampling of Astronaut A

For pre-flight and post-flight sampling the astronaut was provided with swab kits and instructions and collected his own samples; they were stored in the dark at room temperature for a short period before return to Cologne by himself or another ESA staff member, depending on travel plans.

For in-flight sampling, swabs were transported to ISS with the astronaut in the Soyuz capsule in a custom-built protective pouch (Supplementary Fig. S5). A total of 13 sample tubes were provided: 10 for sampling (1 each for the skin and the inside of the suit at the five different sampling sites) and 1 spare in case of damage to a primary tube in transit to ISS, and additional tubes both containing saline, 1 primary and one spare in case of damage to the primary tube. Each ISS-certified EnviroTrans swab rinse kit from Hardy Diagnostics contained 5 ml saline as provided. However, to reduce the total mass uploaded to ISS and to assist the astronaut who would be handling the swabs (and saline) in microgravity, the saline was removed from the 11 sampling tubes prior to transportation in a sterile environment, the swabs returned to the tubes and secured. On board ISS, samples were collected in ESA’s Columbus science module immediately after doffing the suit at the end of the second and final day of wear (Flight Day FD6). In total, the astronaut wore the suit for 13 h (6.0 h on FD5 and 7.0 h on FD6). Between doffing the suit on FD5 and donning it again on FD6, the suit was left out to air. While wearing the SkinSuit, the astronaut performed nominal ISS duties including one 30 min bout of moderate intensity cycle ergometry on FD6. Prior to each swab sample, the astronaut opened the tube containing saline and moistened the tip of the sampling swab. Samples were taken from the same sites as pre-flight, as well as corresponding areas of the inside of the suit. Swabs were then returned to the tubes, secured and placed back in the pouch for return to ground (Supplementary Fig. S5). The pouch returned to ground with the astronaut in the Soyuz capsule at the end of the mission (FD 10) where it was collected from the landing site by ESA staff and transported directly to the laboratory in London, UK. Methods were performed in accordance with relevant regulations and guidelines.

### Skin sample processing

Five body sites corresponding to areas of skin covered during SkinSuit wear were sampled using rayon-tipped Sterilin F155CA swabs or ISS-certified EnviroTrans swab rinse kits containing 5 ml saline (Hardy Diagnostics, Santa Maria, CA): sebaceous (chest; lower back), moist (armpit; groin) and dry (leg) were sampled by gently rubbing the moistened swab tip over a 4 cm^2^ area of skin. In some cases the corresponding area on clothing was sampled. Samples were maintained at room temperature prior to extraction of DNA. The swab tip was removed with sterile scissors, placed in a microcentrifuge tube with 1 ml of B1 buffer (Qiagen, UK), vigorously mixed, 10 µg of lysozyme and 45 µl of proteinase K added and the mixture incubated at 37 °C for 30 min. The swab was removed, the tube briefly centrifuged and the supernatant processed in a FastPrep FP120 cell homogeniser (ThermoFisher, Waltham, MA). After centrifugation, 350 µl of B2 buffer (Qiagen) was added, the mixture incubated at 50 °C for 30 min and purification of genomic DNA attained using QIAGEN Genomic-tip. DNA was precipitated with 0.6 ml isopropanol, centrifuged, washed with cold ethanol and the residue air-dried for 10 min at room temperature. DNA was re-suspended in 30 µl of nuclease-free water and incubated overnight at room temperature. The fluorometric DNA concentration obtained from swabs was generally in the range 100–200 ng/µl. There were differences in the quantity of DNA recovered from different body sites. Those from leg and back regions yielded more DNA than other samples but these variations had no bearing on analyses as all samples were normalised prior to construction of DNA libraries.

### 16S rRNA PCR

The 16S V3/V4 region was amplified by PCR in a final reaction volume of 25 µl using 0.5 µM forward and reverse primers, 10 µl template DNA, and 1x KAPA HiFi HotStart Ready Mix (Anachem, Luton, UK). The primers employed were as described.^49, 50^ Thermal cycling was undertaken at 95 °C for 3 min, followed by 45 cycles at 95 °C for 30 s, 54 °C for 30 s and 72 °C for 30 s, with a final extension step of 5 min at 72 °C. A refrigeration or 'soak' cycle was programmed to end by holding the tubes at 4 °C. PCR products were detected following separation by electrophoresis on 1.2% agarose gels by staining with ethidium bromide. Amplified samples were used for sequencing. Genomic DNA from Microbial Mock Community B (BEI Resources, Manassas, VA), containing genomic DNA from 20 bacterial strains, served as positive controls. Processing of these mock controls, as well as blank negative controls, established that no environmental contamination occurred during sample processing.

### Preparation and sequencing of 16s rRNA MiSeq libraries

16S rRNA amplicon sequencing libraries were prepared using the '16S metagenomic sequencing library preparation' protocol for MiSeq (Illumina, San Diego, CA). Briefly, 16S amplicons were amplified using 16S specific primers with Illumina adaptor sequences for subsequent sample barcoding (forward 5′- TCGTCGGCAGCGTCAGATGTGTATAAGAGACAGCCTACGGGNGGCWGCAG; reverse 5′- GTCTCGTGGGCTCGGAGATGTGTATAAGAGACAGGACTACHVGGGTATCTAATCC). A 10-pM pooled library with PhiX control was analysed using 2× 300 bp Illumina MiSeq V3 cartridges and processed in the Illumina MiSeq.

### Bioinformatics

Sequences were clustered into OTUs at 97% sequence identity using a closed reference method (NINJA-OPS, v. 1.5). The resulting OTUs were analysed using phyloseq in R (v. 1.20.0). Sequences were filtered of error by keeping only OTUs that were present at greater than two reads in 20% of all samples. Before any metrics were computed between samples, all samples were normalised to 1000 reads per sample. Alpha diversity was calculated using Faith’s phylogenetic diversity metric and beta diversity was calculated using Bray–Curtis similarity index. The percent total species represented in the samples collected was estimated using Good’s coverage, and the average coverage across all samples was 86.3% at 0.03 divergence. PERMANOVA and ANOSIM statistical significance measures as well as CCA were calculated using the vegan package in R.

### Availability of data

Fastq files containing DNA sequences have been deposited in the EMBL Nucleotide Sequence Database of the European Nucleotide Agency with accession number PRJEB20777.

## Electronic supplementary material

Supplementary information

Interactive data file S1

Interactive data file S2

Table S1
