# Supplementary material for: Impact of the Mk VI SkinSuit on skin microbiota of terrestrial volunteers and an International Space Station-bound astronaut
Source: NPJ Microgravity. 2017 Sep 7;3:23. doi: 10.1038/s41526-017-0029-5 (PMC5589758; doi:10.1038/s41526-017-0029-5)
Supplement: Supplementary file 1 — Supplementary information [file 41526_2017_29_MOESM1_ESM.docx]

**Supplementary Information**

**Impact of the gravity-loading countermeasure SkinSuit on skin microbiota of terrestrial volunteers and an International Space Station-bound astronaut**

Richard A. Stabler, Helena Rosado, Ronan Doyle, David Negus, Philip A. Carvil, Juan G. Kristjánsson, David A. Green, Rafael Franco-Cendejas, Cadi Davies, Andreas Mogensen, Jonathan Scott and Peter W. Taylor

**Interactive Data files**

**S1.** Consolidated OTU data for dry immersion volunteers: volunteers #45, #275, #392, #477, #636.

A, armpit; B, back; C, chest; G, groin. 1, pre gym kit; 2, post gym kit; 3, pre SkinSuit; 4, post SkinSuit wear.

**S2.** Consolidated OTU data for Astronaut A: A, armpit; B, back; C, chest; G, groin; L, Leg. Sampling points correspond to those indicated in Figure 5.

**Table S1.** Astronaut A: Most frequently identified bacterial Operational Taxonomic Units (OTUs) identified in skin swabs. For each body sampling site (armpit, back, chest, leg, groin) the twenty most common OTUs were ranked according to abundance (1 = most abundant [red], 20 = least abundant [green]) across all sample time points. Staphylococcal OTUs without GreenGenes species level taxonomy information were analysed by BLAST. Column ‘BLAST’ identifies the highest BLAST match using the full 16S sequence to a named bacterial species. BLAST statistics are given in ‘Max score, Query cover, E value, Identity’.


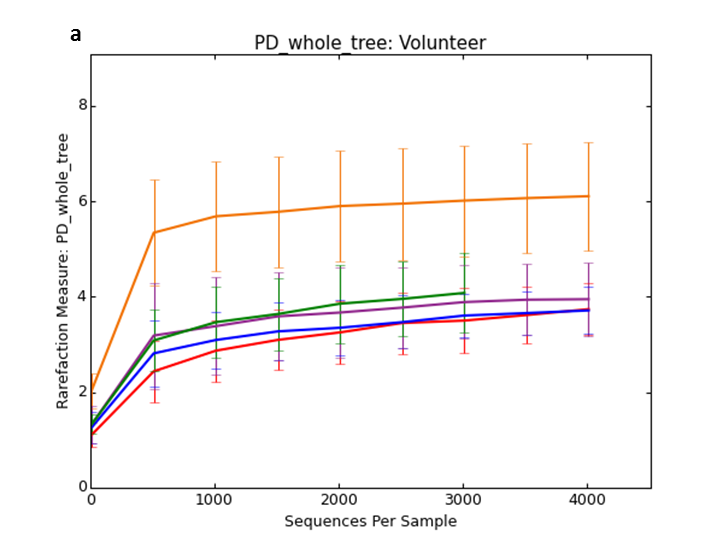


**
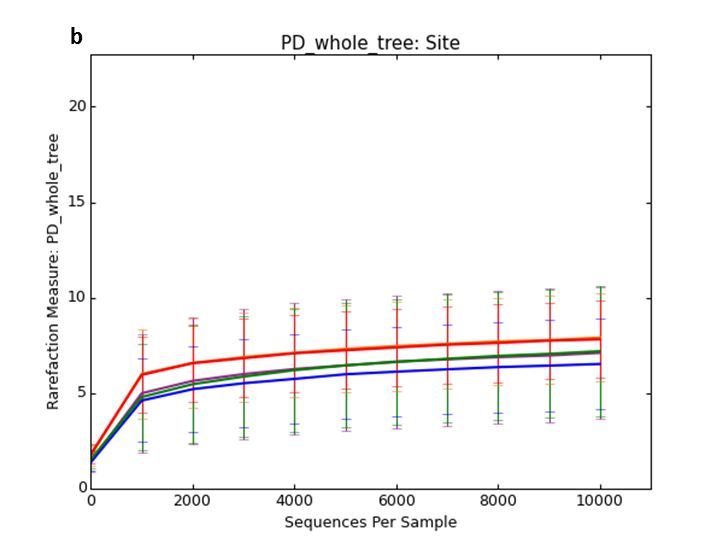
**

**Figure S1.** Rarefaction analysis of skin microbiota of human volunteers undergoing dry immersion. Alpha diversity is represented by phylogenetic differences between individual volunteers. (**a**) Data from armpit, back, groin and chest swabs pooled for each volunteer. Volunteer #45, red; #275, blue; #392, orange; #477, green; #636, purple. (**b**) Pooled data for each site from the five volunteers. Armpit, red; back, blue; chest, orange; groin, green.


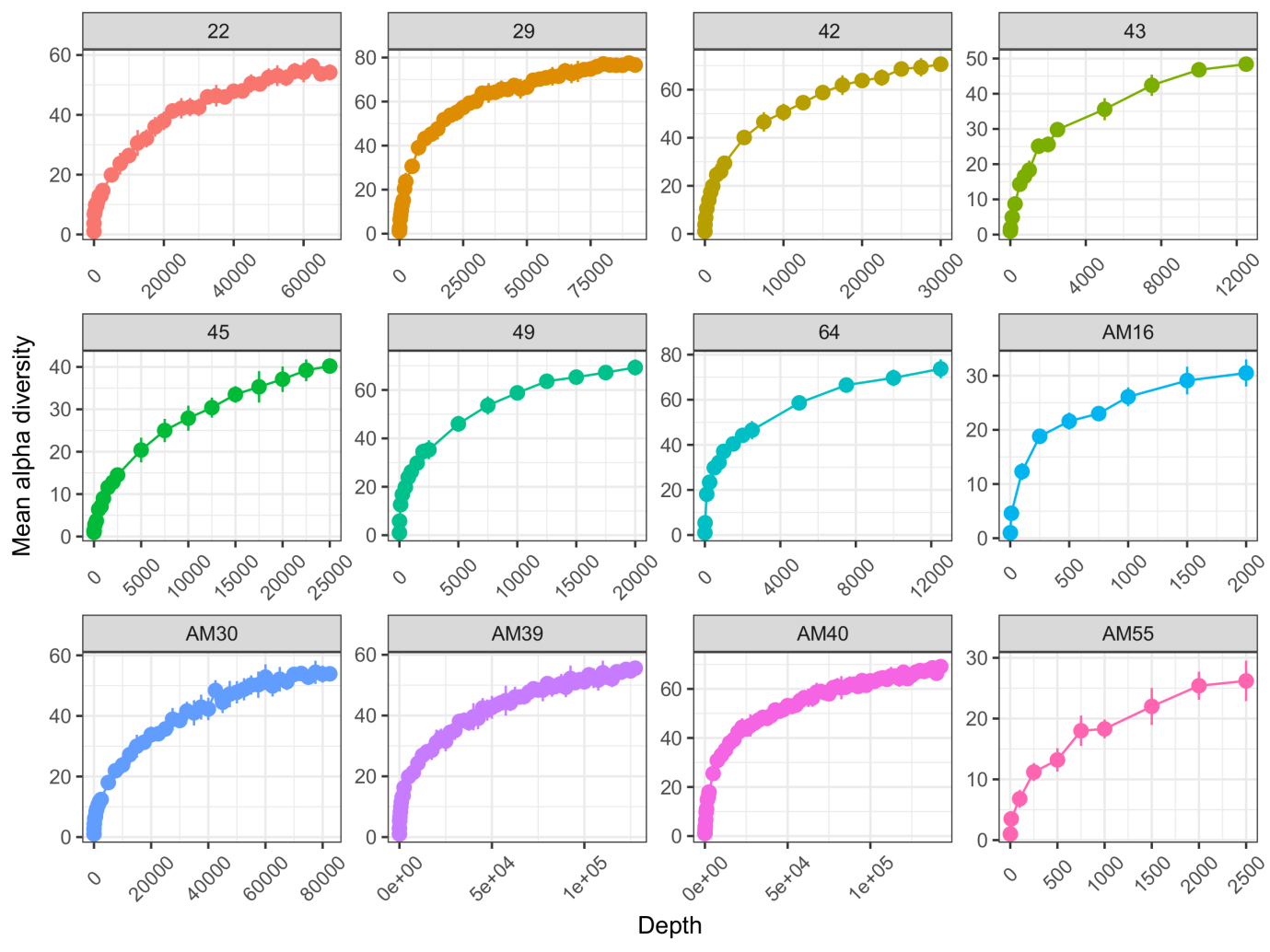


**Figure S2.** Rarefaction analysis of skin microbiota of the 12 samples with the lowest Good’s coverage scores. Alpha diversity is represented by mean number of OTUs observed in the sample after 10 repeated random subsamples were taken at each depth. Sample 22 had a Good’s coverage score of 0.68, 29 = 0.68, 42 = 0.68, 43 = 0.68, 45 = 0.52, 49 = 0.67, 64 = 0.65, AM16 = 0.62, AM30 = 0.64, AM39 = 0.66, AM40 = 0.67, AM55 = 0.59.


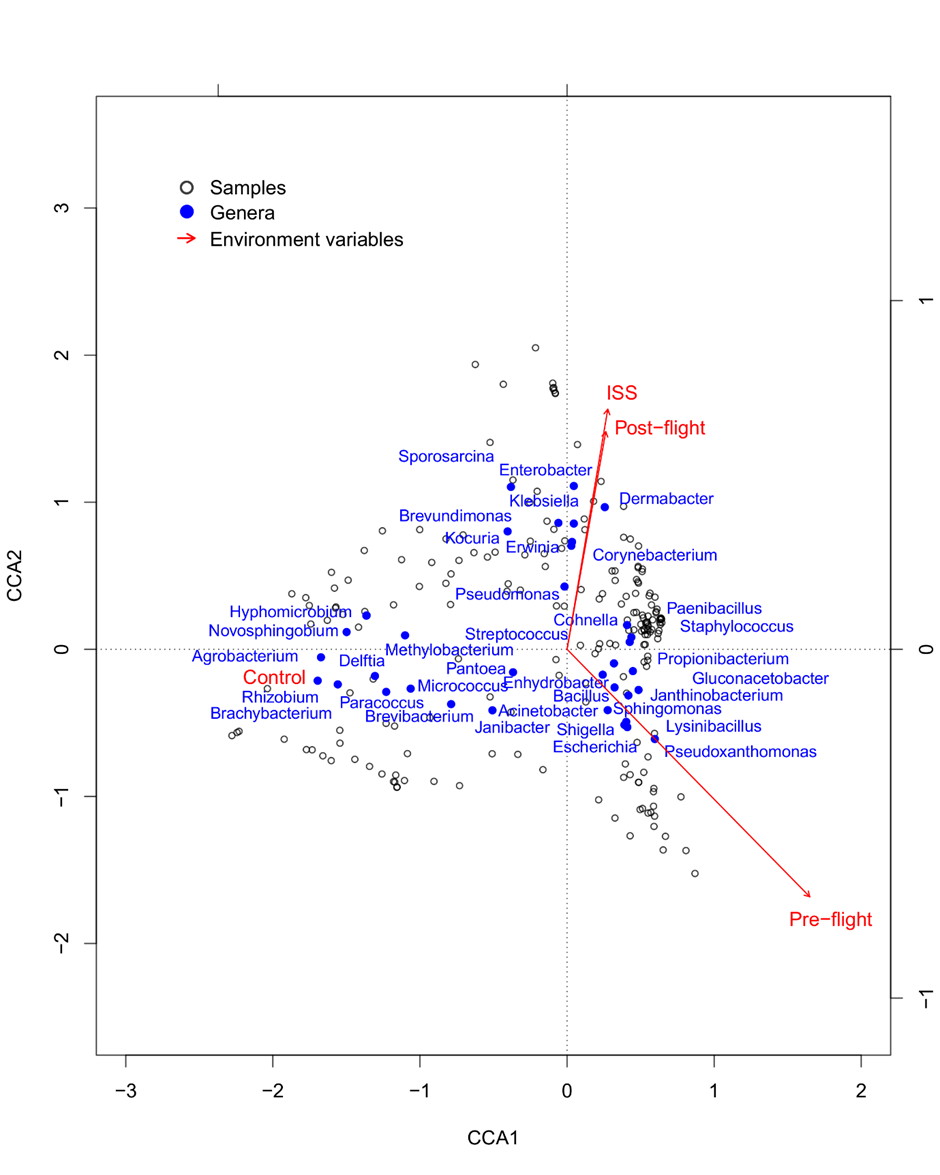


**Figure S3.** Canonical Correspondence Analysis (CCA) showing environment variables of samples were collected from Astronaut A pre-flight, post-flight, on the ISS and from one of the volunteers (Control) in relation to certain abundances of relevant genera.


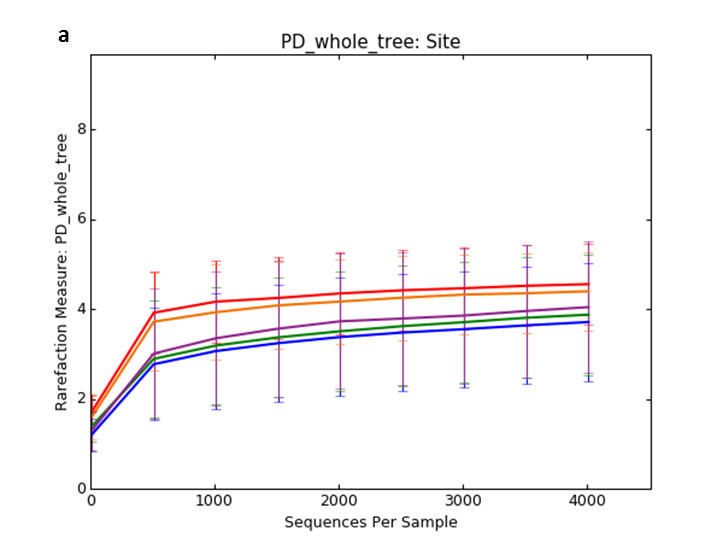


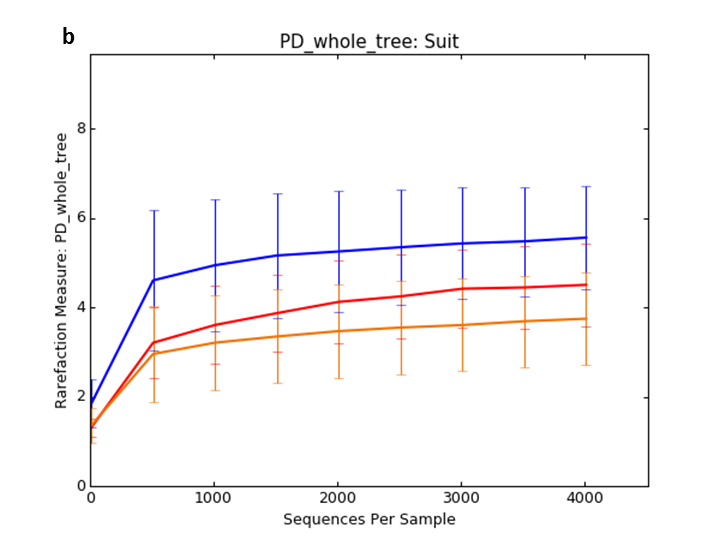


**Figure S4.** Rarefaction analysis of skin microbiota of Astronaut A. (**a**) Data from all armpit (red), back (blue), groin (green), chest (orange) and leg (purple) swabs. (**b**) Pooled data for pre-flight (orange), ISS (red) and post-flight (blue) phases of the mission.


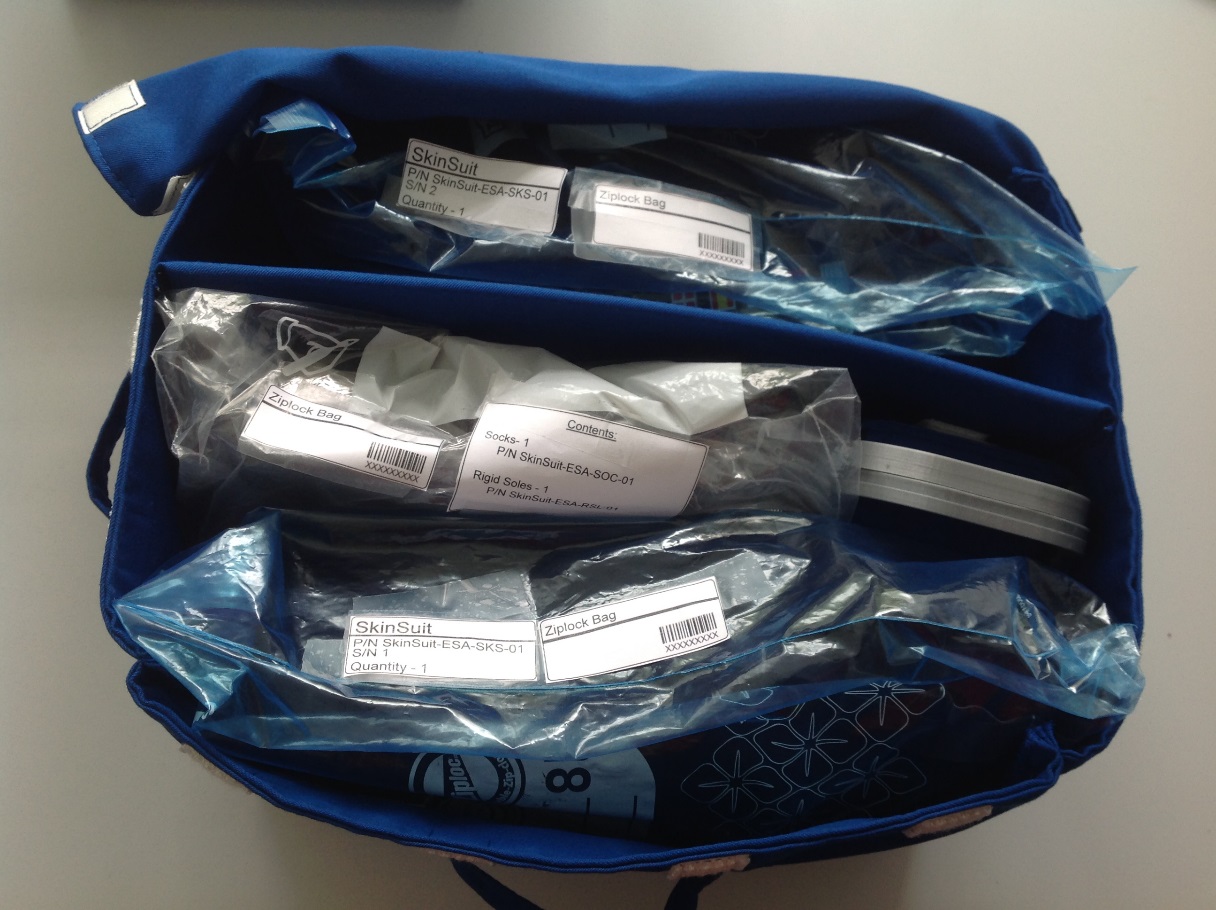


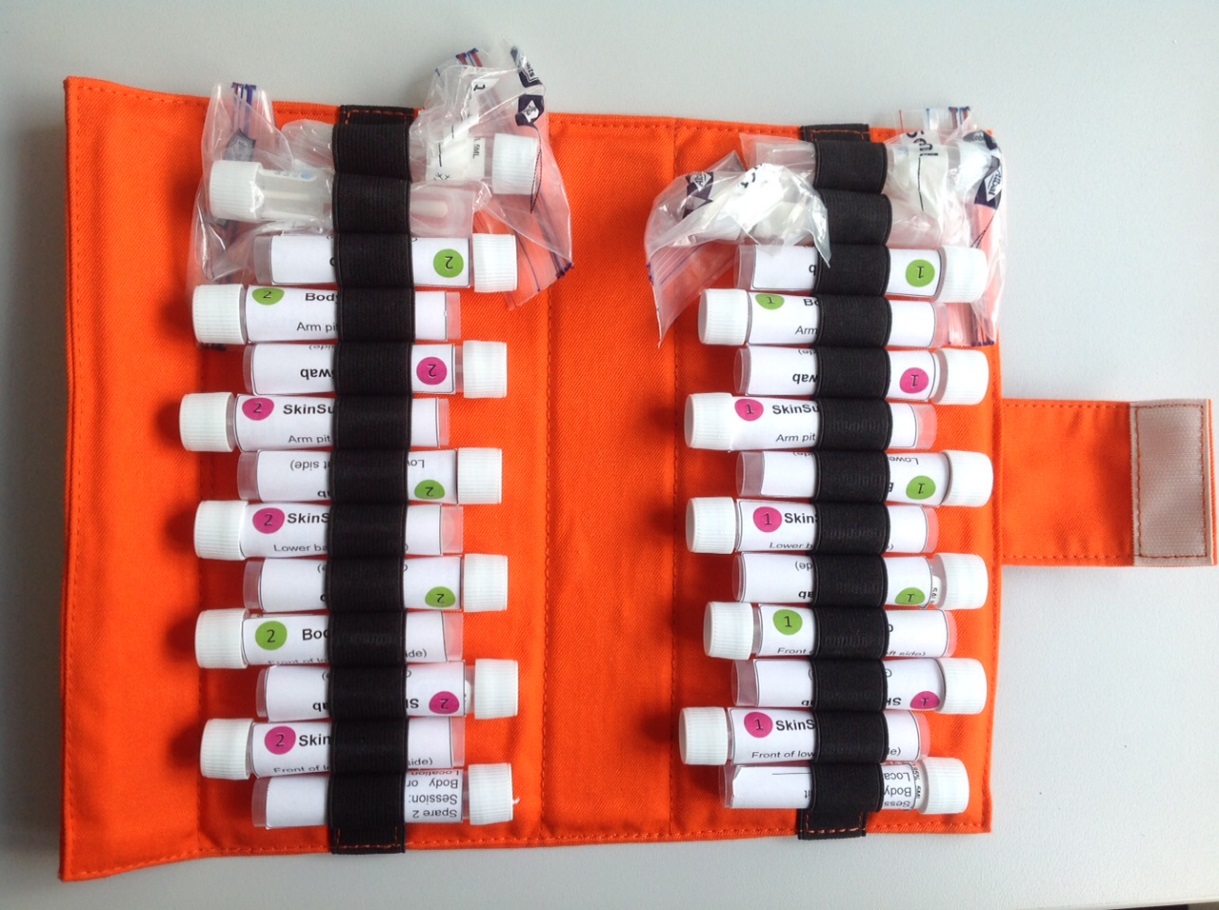


**Figure S5.** Sampling kit for use on ISS by Astronaut A. Upper image shows the custom-built pouch used to transport sample kits to the space station in the Soyuz capsule; the lower image shows the opened sampling kit.
